# OpenQPAIData: Measurements of test objects with reference labels for quantitative photoacoustic imaging research

**DOI:** 10.1038/s41597-026-08004-6

**Published:** 2026-08-04

**Authors:** Janek Gröhl, Sandeep Kumar Kalva, Berkan Lafci, Francesca Di Cecio, Ran Tao, Thomas R. Else, Lorna Wright, Daniel Razansky, Sarah E. Bohndiek

**Affiliations:** 1 Institute for Experimental Molecular Imaging, RWTH Aachen University Hospital, Aachen, Germany; 2 Department of Physics, University of Cambridge, Cambridge, United Kingdom; 3 Cancer Research UK Cambridge Institute, University of Cambridge, Cambridge, United Kingdom; 4 Institute of Pharmacology and Toxicology and Institute for Biomedical Engineering, Faculty of Medicine, University of Zurich, Zurich, Switzerland; 5 Institute for Biomedical Engineering, Department of Information Technology and Electrical Engineering, ETH Zurich, Switzerland; 6 Department of Biosciences and Bioengineering, Indian Institute of Technology Bombay, Powai, Mumbai, 400076 India; 7 Department of Bioengineering, Imperial College London, London, United Kingdom

## Abstract

The goal of quantitative photoacoustic imaging (qPAI) is to determine absolute chromophore concentrations from multispectral photoacoustic images. Achieving this goal would enable bias-free molecular photoacoustic imaging and the derivation of functional tissue biomarkers, such as local blood oxygenation, with numerous clinical implications. To deliver qPAI, two inverse problems must be solved: the *acoustic inverse problem* of reconstructing the initial pressure distribution from measured signals, and the *optical inverse problem* of reconstructing spatial images of optical absorption from the initial pressure. Unfortunately, these inverse problems are difficult to solve in practice. The validation of proposed qPAI algorithms is particularly challenging due to the lack of experimentally measured ground truth of the optical and acoustic tissue properties. With *OpenQPAIData*, we present a dataset of 30 tissue-mimicking phantoms imaged using three different photoacoustic systems: the Multispectral Optoacoustic Tomography (MSOT) InVision commercially produced system from iThera Medical GmbH, and the custom-built Transmission Reflection Optoacoustic and Ultrasound (TROPUS) and Spiral Volumetric Optoacoustic Tomography (SVOT) imaging systems developed by Prof. Daniel Razansky’s group. The dataset includes raw measurements, reconstructed images, manually segmented reference annotations, and matched optical property maps derived from double-integrating-sphere (DIS) measurements. By providing these multi-device imaging data with reference optical properties, we believe that *OpenQPAIData* enables the benchmarking of both acoustic and optical inverse solvers and supports the development of image quality assessment metrics for photoacoustic imaging.

## Background & Summary

Photoacoustic imaging (PAI, also referred to as optoacoustic imaging) is a non-invasive biomedical imaging modality that combines the advantages of optical imaging and ultrasound to produce high-contrast, high-resolution images deep within biological tissues. PAI relies on the photoacoustic effect, where pulsed laser light is absorbed by light-absorbing molecules, leading to a rise in temperature and subsequent thermal expansion, which generates sound detectable with broadband detectors. Typically, ultrasound transducers or Fabry-Pérot interferometers are used and placed on the tissue surface^1^. The detected signals are processed using reconstruction algorithms to produce images of the absorbed light energy in the tissue and can provide information about the molecular composition of the tissue^2^. Being a multi-physics imaging modality, PAI can image deeper tissues compared to purely optical imaging methods by exploiting the lower scattering of ultrasound waves in tissue compared to light^1^. Given its potential to provide high molecular contrast and derive functional tissue properties such as blood oxygenation levels^3^, PAI has already found potential exciting clinical applications^4^ in the imaging of breast cancer^5^, inflammatory diseases^6^, neuromuscular diseases^7^, and skin diseases^8^.

To enable quantitative photoacoustic imaging (qPAI), such as molecular concentration information, two inverse problems need to be solved^9^. The first is the *acoustic inverse problem*, which is concerned with reconstructing the initial pressure distribution p_0_ from the measured time series data. The second is the *optical inverse problem*, which is concerned with calculating the optical absorption coefficient from p_0_, by accounting for the light fluence. These problems are both extremely hard to solve^10^. Solving the acoustic inverse problem is precluded by the fact that tissue acoustic properties are typically unknown and that hardware limitations affect the frequency response and noise characteristics of the recorded data. While a limited bandwidth of the frequency response would lead to characteristic limited-data artifacts in the image^11^, noise in the measurement data can lead to a reduced signal-to-noise ratio of the reconstructions. The primary challenges for the optical inverse problem are the absorption/scattering non-uniqueness and the unknown Grüneisen parameter^11^, making the optical inverse problem highly ill-posed. Many promising algorithms have been proposed and validated in simulation studies, but a challenge when translating these methods to experimental measurements is that the ground truth tissue properties are unknown, making an evaluation difficult^12^.

**Fig. 1:**
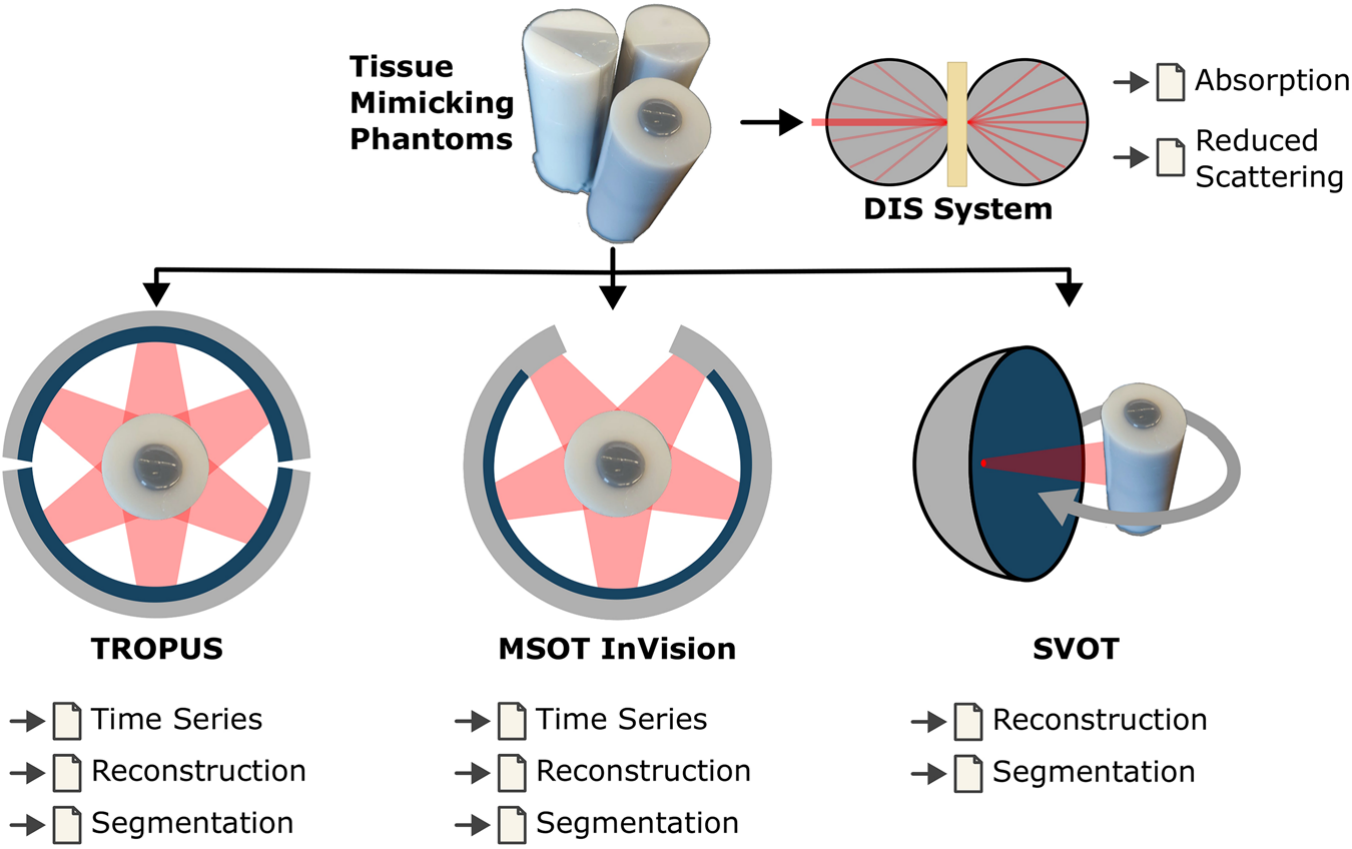
Graphical overview of the dataset. We imaged tissue-mimicking phantoms (photograph top) with three different photoacoustic imaging devices: the Multispectral Optoacoustic Tomography (MSOT) InVision, the Transmission Reflection Optoacoustic and Ultrasound (TROPUS), and the Spiral Volumetric Optoacoustic Tomography (SVOT) system. In addition, we took reference measurements of the optical absorption and reduced scattering coefficients of the phantom materials using a double-integrating-sphere (DIS) system.

Here, we present a dataset that can be used for the testing and validation of novel algorithms for solving both the acoustic and optical inverse problems in PAI^13^. We fabricate N = 30 tissue-mimicking test objects (phantoms) made primarily of mineral oil and polystyrene (also referred to as copolymer-in-oil) and vary their optical properties to cover a range of physiological values. We image these phantoms with three different photoacoustic devices, provide time series measurements, and annotate reference backprojections with independent absorption and scattering values obtained from double-integrating-sphere measurements (Fig. 1). The result is a multi-device dataset that combines time series measurements with knowledge of the optical and acoustic properties of the imaged objects. As such, we enable the end-to-end validation of algorithms that seek to quantify optical absorption and scattering from PAI^14^.

The development of qPAI algorithms also relies on the definition of suitable image quality assessment (IQA) measures. Traditional IQA measures, such as peak signal-to-noise ratio (PSNR) and structural similarity index (SSIM), may not be effective in capturing the nuances of medical images^15–17^. The presented dataset could be used to investigate the usefulness of newly emerging IQA measures in the context of PAI through the comprehensive set of images with corresponding reference semantic annotations. As such, we hope that these data provide a valuable resource for research towards qPAI by enabling the validation of image reconstruction and quantification algorithms.

## Methods

### Phantom fabrication

We fabricated N = 30 tissue-mimicking test objects (phantoms) based on the copolymer-in-oil recipe established by Hacker *et al*.^18^, using the same fabrication process as previously described^14^. The phantoms were designed as cylinders with a diameter of 27.5 mm and a height of 70–80 mm. Each phantom is piecewise constant and consists of a background cylinder into which imaging targets are added. Rectangular samples with a length of 59 mm, a width of 18 mm, and a thickness ranging between 2 mm and 4 mm were prepared from each material for optical characterisation. Sample thicknesses were determined at five distinct locations using digital vernier callipers. The acoustic properties of the phantoms were extensively characterised in prior work^18^. The sound speed of the phantom material at room temperature was measured to be ≈1468 m s^−1^ and the acoustic attenuation was measured to be 4.65 dB cm^−1^Mhz^−1^. The literature-consensus sound speed of the deionised water in the coupling medium is ≈1482 m s^−1^ at 20°C and ≈1496 m s^−1^ at 25°C^19^. The mineral oil base material vendor reports a density of 0.833 g mL^−1^ at 25°C. We assume these values to be nominal for the phantom material, but experimental variability is to be expected. However, as all phantoms were produced by the same person, from the same source material, using a fixed fabrication protocol, we expect the resulting variation across phantoms to have no meaningful impact on downstream analyses.

### Double-integrating-sphere reference measurements

**Fig. 2:**
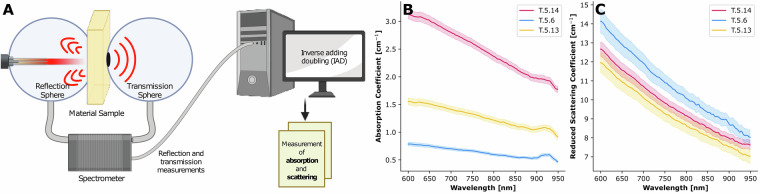
Double-integrating-sphere system schematic and example measurements. (**A**) Simplified schematic of the double-integrating-sphere measurement workflow. (**B**) Exemplar calculation results from three material samples (T.5.6 (low *μ*_*a*_), T.5.13 (medium *μ*_*a*_), T.5.14 (high *μ*_*a*_)) for the absorption coefficient and (**C**) for the reduced scattering coefficient. The median is shown for each sample in a distinct colour, and the standard deviation around the median is shown slightly transparently.

For the measurement of the optical properties of our phantom materials, we use a double-integrating-sphere (DIS) system (Fig. 2), where the total reflection and transmission of the sample are measured using two integrating spheres. The optical absorption and reduced scattering coefficients can then be determined using the inverse adding-doubling (IAD) algorithm^20^. The IAD algorithm uses an initial guess for the optical absorption and reduced scattering coefficients and simulates the corresponding reflection and transmission using the adding-doubling algorithm. The initial guess is iteratively updated using an N-dimensional minimisation algorithm based on the downhill simplex method^21^. During the forward computation, the algorithm uses sphere corrections that account for the non-linear response of integrating spheres to the presence of a sample^22^. In each iteration step, the simulated measurements are compared against the actual measured data until the error is below a certain threshold. To account for the light loss at the sample boundary, the algorithm uses Monte Carlo iterations, which have been shown to increase estimation accuracy at the cost of computational speed^22^.

### Multi-device imaging

**Fig. 3:**
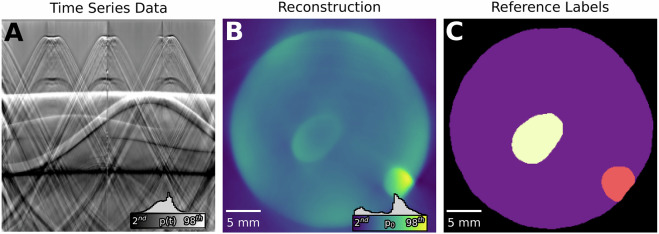
Visualisation of phantom P.5.6.2 with the InVision 256-TF imaging system. (**A**) Raw time series data. The x-axis of the time series data represents different detector elements, and the y-axis represents time. (**B**) A reference reconstruction of the initial pressure distribution. The imaging data are clipped between the 2nd and the 98th percentile to enhance the contrast. (**C**) Hand-annotated reference labels. The images include intensity value distributions as histograms on the bottom right. In the histogram labels, p(t) denotes the acoustic pressure signal amplitude, and p_0_ denotes the initial pressure.

**Fig. 4:**
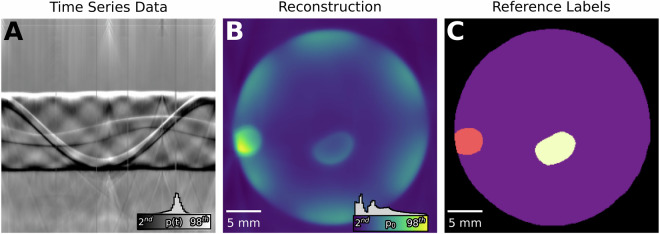
Visualisation of phantom P.5.6.2 with the TROPUS imaging system. The figure panels show (from left to right) the raw time series data (**A**), a reference reconstruction of the initial pressure distribution (**B**), and hand-annotated reference labels (**C**). The imaging data are clipped between the 2nd and the 98th percentile to enhance the contrast. The colour maps show the grey-value distributions inside the images as histograms. The x-axis of the time series data represents different detector elements, and the y-axis represents time. In the histogram labels, p(t) denotes the acoustic pressure signal amplitude, and p_0_ denotes the initial pressure.

**Fig. 5:**
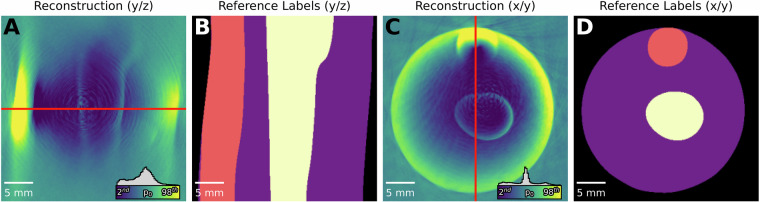
Visualisation of phantom P.5.6.2 with the SVOT imaging system. The figure panels show reference reconstructions of the initial pressure distribution within two orthogonal cross-sections through the 3D volume (**A** and **C**) and hand-annotated reference labels (**B** and **D**). The red lines in the reconstructions indicate the location of the other cross section. The imaging data are clipped between the 2nd and the 98th percentile to enhance the contrast. The colour maps show the image intensity as histograms. In the histogram labels, p_0_ denotes the initial pressure.

The phantoms were imaged using three different devices: the MSOT InVision 256-TF (iThera Medical GmbH, Germany)^23^, the Transmission Reflection Optoacoustic and Ultrasound (TROPUS) imaging system^24^, and the Spiral Volumetric Optoacoustic Tomography (SVOT) imaging system^25^. Here, we describe these systems in brief, and full technical descriptions can be found in the cited references. The phantom shown in the reference images (Figs. 3, 4, and 5) is the phantom with ID P.5.6.2. At 800 nm, the background phantom material has *μ*_*a*_ = 0.4 cm^−1^ and ${\mu }_{s}^{{\prime} }=6.5\,{{\rm{cm}}}^{-1}$, the center absorber has *μ*_*a*_ = 3.5 cm^−1^ and ${\mu }_{s}^{{\prime} }=4.8\,{{\rm{cm}}}^{-1}$, and the superficial absorber has *μ*_*a*_ = 1.2 cm^−1^ and ${\mu }_{s}^{{\prime} }=8.7\,{{\rm{cm}}}^{-1}$.

#### The InVision imaging system

The MSOT InVision 256-TF system is a tomographic system that captures 2D cross-sectional imaging slices. It has 256 piezoelectric ultrasound detection elements with a centre frequency of 5 MHz and a bandwidth of 60% that cover a total angle of 270°. The laser energy is measured for each pulse by the system, and we averaged over 10 consecutive frames to minimise thermal noise and laser power variability. We imaged a single tomographic slice at five wavelengths (700, 750, 800, 850, 900 nm) and recorded device-internal measurements of the laser pulse energy. We modelled the laser outputs as slits with a slit length of 6.12 mm. They were placed at a radial distance of 37 mm from the centre and 12.37 mm from the imaging plane. They were tilted by 23.8° towards the centre and had an opening angle with a Gaussian standard deviation of 5.5°. There were ten total laser outputs, five equally spaced outputs, placed at 72° intervals on both sides of the imaging plane. The example time series is shown after pulse energy correction and DC offset correction (Fig. 3).

#### The TROPUS imaging system

The TROPUS system is a tomographic system with 512 detection elements, each with a centre frequency of 5 MHz and a bandwidth of 60% that cover a total angle of 348°. We manually measured the laser energy of the laser (nanosecond Nd:YAG laser with pumped source of 15 Hz repetition rate (Spectra-Physics, Santa Clara, CA, USA)) and also averaged over 10 consecutive frames. We imaged a single tomographic slice at five wavelengths (700, 750, 800, 850, 900 nm) and used a power meter before the experiments to record the laser power spectrum over the target wavelength range. We modelled the laser outputs as slits with a slit length of 4.325 mm. They were placed at a radial distance of 40 mm from the centre and 12.5 mm from the imaging plane. They were tilted by 24° towards the centre and had an opening angle with a Gaussian standard deviation of 5.2°. There were twelve total laser outputs, six equally spaced outputs, placed at 60° intervals on both sides of the imaging plane. The example time series is shown after pulse energy correction and DC offset correction (Fig. 4).

#### The SVOT imaging system

The Spiral Volumetric Optoacoustic Tomography (SVOT) system employs a hemispherical transducer with 512 elements (centre frequency 7 MHz, bandwidth 85%), which is rotated around the object to collect true 3D tomographic data. We used the same laser as in the TROPUS system and imaged at five wavelengths (700, 740, 800, 840, 900 nm) and corrected the measurement for the laser pulse energy. To reconstruct 3D images of the phantoms, we imaged at multiple locations in 18° intervals around the phantom, moving the transducer with a motorised stage. The laser source of the SVOT is described to have a Gaussian illumination profile with a size of ≈10 mm at the full width at half maximum on the object surface^26^.

### Reference Reconstructions

We reconstructed TROPUS and InVision measurements using an in-house implementation of the filtered backprojection algorithm^27^, which is accessible open source within the PATATO toolbox^28^. We decided to use this algorithm as it is widely used and its limitations are well understood. It was recently also shown that the filtered backprojection implemented in PATATO yields surprisingly accurate reconstructions for this particular detection geometry^29^. For the 2D imaging setting, we reconstructed the images into a 300 × 300 pixel grid representing an area of 32 × 32 mm^2^. For the SVOT images, we reconstructed a volume of 32 × 32 × 32 mm^3^ into a 360 × 360 × 360 voxel grid. The PATATO implementation applies the backprojection algorithm to the time derivative of the detected acoustic signals after applying a bandpass filter between 5 kHz and 7 MHz to partially account for the frequency response of the imaging system. The SVOT measurements were reconstructed with a custom MATLAB 3D back-projection implementation, where each detection element is further split into equally spaced sub-elements before reconstruction^30^. A technical description of the SVOT transducer, including the frequency response, can be found in prior work of Deán-Ben *et al*.^31^. We assume a sound speed of 1489 m/s for image reconstruction in the InVision system and 1480 m/s for the TROPUS system, due to differences in the temperature of the coupling medium. We assume that other temperature-dependent acoustic property changes are negligible compared to the overall experimental uncertainties.

### Digital Twin Construction

We manually segmented the reference MSOT and TROPUS reconstructions using the Medical Imaging Interaction Toolkit (MITK)^32^. We used a semi-automated segmentation scheme for the 3D SVOT annotations. In the first step, several 2D cross-sectional images along each of the three dimensions were annotated manually. Subsequently, we employed an interpolation scheme to fill in the blanks across each dimension and averaged the results for each dimension into a final segmentation mask. For phantoms with a strong symmetry along the z-dimension, we only annotated 2D cross-sectional images along the z-dimension and interpolated between these. Specifically, we interpolated the hand-drawn label masks along the x, y, and z axes using a custom linear interpolation function to fill in the missing gaps. We then averaged the resulting direction-specific segmentation volumes to reduce directional bias. We smoothed the volume using a Gaussian filter with an anisotropic filter kernel, where the kernel sizes were tuned to give the best segmentation result for each phantom. Finally, we binarised the volumes using a threshold of 0.5. We annotated each segmented area with reference absorption and scattering coefficients. For the simulation of photoacoustic signals, digital twins of the TROPUS and InVision devices, including their illumination and detection geometry, are available through the SIMPA simulation tool^33^.

## Data Record

All data can be found open access under a Creative Commons CC-BY license on Zenodo 10.5281/zenodo.14044853^13^. The data is organised as follows:

**DIS** Contains the double-integrating-sphere measurements of samples prepared from the same material that was used to cast the phantoms. The *raw* data folder contains the raw data measurements (minimum reflectance, maximum reflectance, minimum transmittance, maximum transmittance), and sample measurements, such as information on the exact measurements of the phantom material constituents in the *recipe.txt* file and calliper thickness measurements of the sample in the *thickness.txt* files. The *processed* data folder contains the results of the inverse adding-doubling procedure and, as such, the wavelength-dependent absorption and scattering properties of the material samples. For a phantom of name X, the data can be found in a numpy array in the folder *DIS/processed/X.a/X.npz*.

**invision** Contains all data associated with the MSOT InVision 256-TF device. The *uncorrected* time series measurement data are in the *raw* data folder in the IPASC data format^34^, which can be read using the PACFISH tool (https://github.com/IPASC/PACFISH). The *recon* folder contains back-projected measurements in the form of a NumPy data array. The *labels* folder contains manual segmentations of the different phantom materials drawn onto the back-projected images. The *mua* and *musp* folders contain the optical absorption (*μ*_*a*_) and reduced scattering (${\mu }_{s}^{{\prime} }$) coefficients mapped into the manual labels in units of cm^−1^.

**tropus** Contains all data associated with the TROPUS device. The internal folder and data structure are the same as described for the *invision* folder.

**svot** Contains all data associated with the SVOT device. In comparison to the other two devices, the *raw* data cannot be shared, due to the data size, so only the reference reconstructions are made available. In addition, there is a *labels_manual* folder containing all of the 2D manually annotated slices of the 3D images, whereas the *labels* folder contains the fully-segmented 3D images, which are the result of the interpolation algorithm described above. We decided to share the SVOT data, as they add valuable information on the 3D context of the 2D tomographic images of the TROPUS and InVision systems.

**material_mapping.json** This file contains the mapping of the labels of the segmented images to the correct material samples for that region, which allows for the mapping of *μ*_*a*_ and ${\mu }_{s}^{{\prime} }$ values to the segmentation masks.

Every phantom is assigned a unique name that starts with a “P”, which is kept across devices and throughout the entire file structure. Material samples are also assigned unique names, starting with a “T”. In this dataset, the naming convention is P.5.X and T.5.X, where X is a unique reference to the phantom or material.

## Technical Validation

### Photoacoustic measurement devices

Technical validation of the photoacoustic measurement devices was not part of acquiring this dataset. Nonetheless, the imaging systems used were rigorously validated previously. The MSOT InVision 256-TF device was commercially available and manufactured by iThera Medical GmbH (Munich, Germany). The device we used in this study was purchased in 2013 and has been used in numerous in vivo studies^35–37^. Importantly, Joseph *et al*.^23^ performed a technical validation study to evaluate the precision of this exact system in phantoms and in vivo in mice. The system has been used robustly in a series of subsequent phantom studies^12,38^. The TROPUS imaging system is a research system, which was carefully characterised and used in several in vivo mouse studies^24,39,40^. Each phantom was scanned only once per device, as we know from prior studies that the repeatability of these systems is high (e.g., MSOT intensity error <5%^23^).

### Data Annotation

We manually segmented the phantom regions corresponding to different materials from the reconstructed photoacoustic images. The manual segmentation was feasible since the materials were piecewise constant and expressed sharp boundaries between the material regions. To ensure high confidence in the segmentations, we employed a correction step, where a second person reviewed the annotations and implemented edits to the original segmentation masks.

### Optical Reference Measurements

We cross-validated the accuracy of the DIS measurements with commercially available optical calibration phantoms (BioPixS Ltd, Cork, Ireland), which were characterised using a time-domain system. We measured the wall reflectance of the spheres to accurately account for it in the inverse adding-doubling algorithm, and minimised the amount of back reflection from the sphere casing with a masking tape. These measures lead to an error of less than 2.5% in the estimated *μ*_*a*_ and $\mu {\prime}$ across 16 different calibration phantoms^41^.

**Fig. 6:**
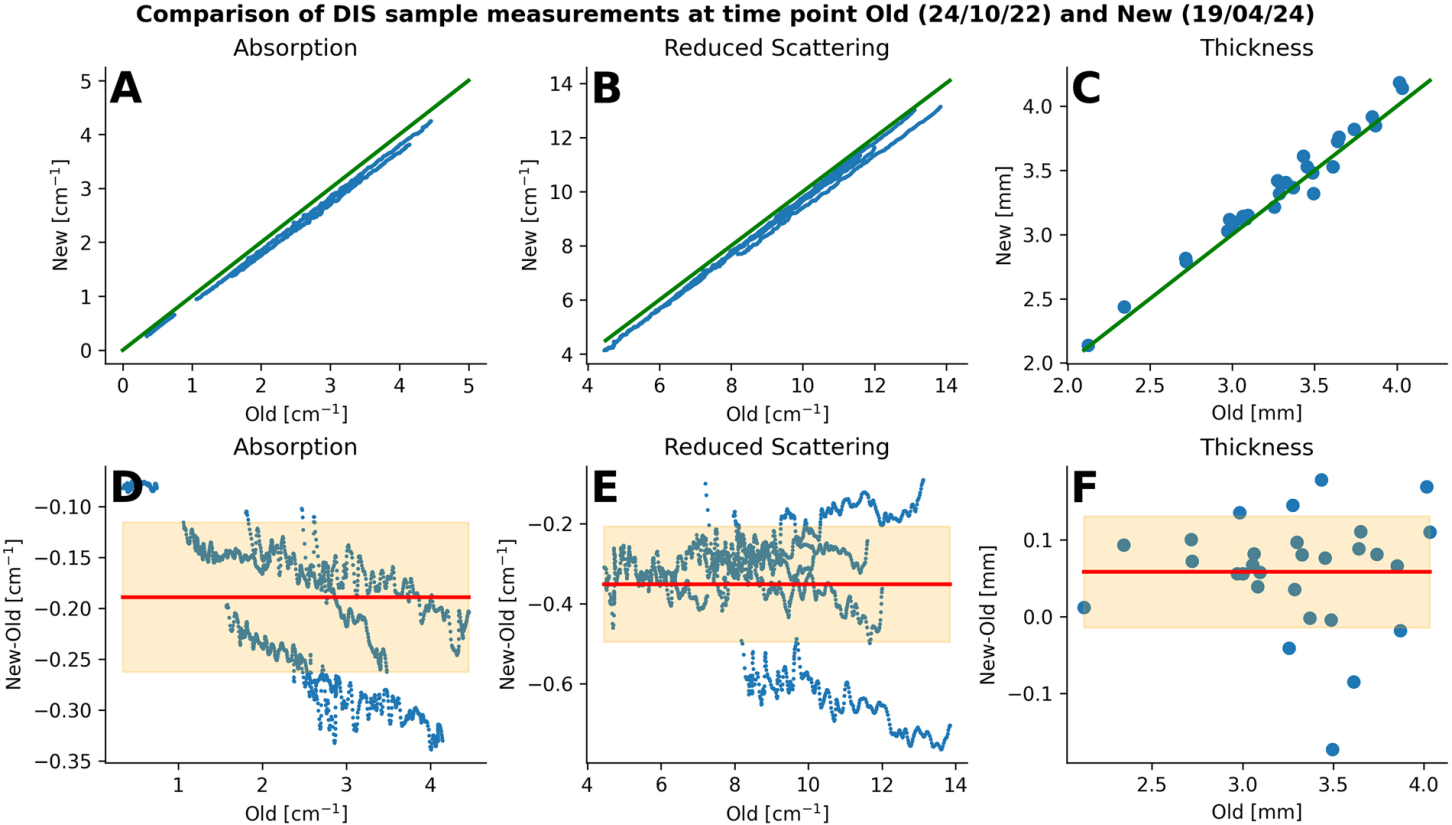
Comparison of sample characterisation results 18 months apart. The upper row shows scatter plots of the old and new estimated sample absorption (**A**), reduced scattering (**B**) and calliper thickness measurements (**C**). The identity line is shown in green. The lower row shows a Bland-Altman-style visualisation of the difference versus the old measurements of absorption (**D**), reduced scattering (**E**), and thickness (**F**). The mean difference is shown in red, and the standard deviation around the mean is highlighted in orange. We used all available material samples for this comparison.

To ensure the repeatability of the measurements, we repeated the DIS measurements 18 months after initial characterisation. The results showed excellent correlation between the two measurement time points with only minor systematic deviations between the measurements (*μ*_*a*_: − 0.19 cm^−1^ and ${\mu }_{s}^{{\prime} }:-0.35\,{{\rm{cm}}}^{-1}$) (cf. Fig. 6). We re-took the manual calliper thickness measurements and found a systematic offset of 0.06 mm, which is likely to explain the systematic differences in the optical property estimates. When correcting for the systematic offset in thickness (but keeping the variability), the systematic error reduces to (*μ*_*a*_: − 0.15 cm^−1^ and ${\mu }_{s}^{{\prime} }:-0.12\,{{\rm{cm}}}^{-1}$). We were positively surprised to find that the material was that stable for such a long time, since no special precautions against photo-bleaching or other sources of sample degradation were taken. The samples were stored in a transparent plastic box on an open shelf in the lab area. Each sample was sandwiched between two glass microscopy slides. The background absorption coefficients range from 0.17 cm^−1^ to 0.77 cm^−1^ and the imaging target coefficients range from 0.8 cm^−1^ to 4.4 cm^−1^. The reduced scattering coefficient for all materials was between 4.3 cm^−1^ to 14.1 cm^−1^.

## Usage Notes and Limitations

The dataset also allows for the simulation of a reference for the initial pressure distribution from the digital phantom twin for assessing the performance of acoustic inversion algorithms, as we have shown in prior work for the InVision data-subset^29^. Since the numerical model hinges on many parameters that have to be approximated, we did not feel comfortable sharing simulations which would then be treated as *de facto* ground truth as part of this dataset. The SIMPA toolkit^33^ provides a complete digital twin implementation of the InVision. Furthermore, the acoustic detector specifications for both the InVision and TROPUS systems can be found in the IPASC file format, and respective implementations of the illumination profiles can be found on a custom fork of mcx (https://github.com/jgroehl/mcx/).

We have not characterised the Grüneisen parameter of the phantom material and dyes that we are using. For numerical modelling purposes, we assume that it is constant and ≈0.8 for mineral oil^42^. It should be kept in mind, however, that the Grüneisen parameter is known to be temperature and concentration dependent. The water temperature of the InVision measurements was at 25°C, whereas the TROPUS and SVOT measurements were taken at room temperature (approximately 21°C). Because of this, there will be slight mismatches in the Grüneisen parameter and other temperature-dependent effects between the measurements. There was no way to circumvent this practically at present, as the InVision does not support temperatures below 25°C. We assume the acoustic attenuation and any fluorescence or other optical processes in the nigrosin dye to be negligible.

We have not characterised the scattering anisotropy of the phantom material with the titanium dioxide particles. From other literature, we assume that the anisotropy is 0.7, which is typical for titanium dioxide scatterers in tissue phantoms^43,44^. We also did not characterise the refractive index of the material and assumed a refractive index of 1.41 based on other literature^45^.

The illumination geometries of both the TROPUS and InVision systems have been optimised to produce homogeneous radiant exposure for phantoms with a diameter of ≈20 mm (cf. e.g. Fig. 3 in the paper by Merčep *et al*.^39^). The phantoms used for this study were significantly larger (diameter of 27.5 mm) and, as such, significant surface variation of the radiant exposure can be observed.

Because of excessive artifacts in one of the SVOT phantom reconstructions, we can only share 29 SVOT phantom measurements, and phantom P.5.8.3 is missing. The artifacts were caused by air inclusions inside the phantom that did not visibly affect the 2D InVision and TROPUS measurements. Further, the centre imaging planes were not standardised between the three modalities and were chosen based on experimental constraints, hence imaging data from the three systems are not necessarily directly comparable. We also attempted no cross-device normalisation of PA amplitudes, and all data are supplied in arbitrary units after energy correction.

The segmentation masks were hand-drawn based on the reference reconstruction. While the phantom has sharp boundaries between the material interfaces, the photoacoustic image reconstructions could be quite blurry at times, possibly caused by out-of-plane effects, introducing uncertainty in the outlines. Through the correction step involving a second annotator, we are confident that the segmentation quality is adequate for the reference reconstructions and we have already successfully used the references both for pixel-wise regression^14^, as well as for forward simulation and pixel-wise image quality assessment^29^. Nevertheless, there may be slight differences in the object boundaries when employing different reconstruction algorithms. In these cases, the users should corroborate the adequacy of our provided segmentation masks through careful visualisation and correct for mismatches.

## Data Availability

The data is available on Zenodo 10.5281/zenodo.14044853 under a CC-BY-4.0 license.

## Code availability

Python codes to reproduce the visualisations of this paper, as well as minimally working example codes on how to access the items in the data record, can be found on GitHub: https://github.com/BohndiekLab/OpenQPAIData. They are available under an MIT license.
